# On Blurry Boundaries When Defining Digital Biomarkers: How Much Biology Needs to Be in a Digital Biomarker?

**DOI:** 10.3389/fpsyt.2021.740292

**Published:** 2021-09-30

**Authors:** Christian Montag, Jon D. Elhai, Paul Dagum

**Affiliations:** ^1^Department of Molecular Psychology, Institute of Psychology and Education, Ulm University, Ulm, Germany; ^2^Department of Psychology, University of Toledo, Toledo, OH, United States; ^3^Department of Psychiatry, University of Toledo, Toledo, OH, United States; ^4^Applied Cognition, Los Altos, CA, United States

**Keywords:** digital phenotyping, mobile sensing, digital biomarkers, psychology, medicine, psychiatry

## Abstract

Recent years have seen a rise in research where so called “digital biomarkers” represent the focal study interest. Many researchers understand that digital biomarkers describe digital footprints providing insights into healthy and pathological human (neuro-)biology. Beyond that the term digital biomarker is also used at times to describe more general concepts such as linking digital footprints to human behavior (which itself can be described as the result of a biological system). Given the lack of consensus on how to define a digital biomarker, the present short mini-review provides i) an overview on various definitions and ii) distinguishes between direct (narrow) or indirect (broad) concepts of digital biomarkers. From our perspective, digital biomarkers meant as a more direct (or narrow) concept describe digital footprints being directly linked to biological variables, such as stemming from molecular genetics, epigenetics, endocrinology, immunology or brain imaging, to name a few. More indirect concepts of digital biomarkers encompass digital footprints being linked to human behavior that may act as latent variables indirectly linked to biological variables.

## Background

With the rise of the smartphone and Internet of Things, human societies are rapidly moving toward a globally connected world (1–3). This digitalization led to improvements in many areas of human society, including easier and faster communication *via* far distances. Despite the many advantages of a totally connected world, there are also downsides, where it becomes visible that developments in a digital society go along with (un-)intended negative side effects (4). For instance, in a totally connected digital world humans leave digital footprints everywhere they go. Such ubiquitously available data easily can lead to privacy problems and manipulations of human behavior (5), for instance *via* psychological targeting (6, 7). This said, the aforementioned digital footprints can also improve the psychological and medical sciences (8–12) by providing scientists and practitioners alike the chance to sense psychological/medical states via mobile devices such as the smartphone (“mobile sensing”) or to phenotype such health conditions via digital footprints [“digital phenotyping”; (13)], but of course data protection issues also arise (14, 15). Of interest, beyond mobile sensing and digital phenotyping the term digital biomarker is increasingly found in the literature (16–18). Therefore, we try to clarify in the present mini-review what is actually meant when researchers speak of a digital biomarker. For this purpose, we revisited several recent papers, where scientists also prominently used the term digital biomarker in their paper titles.

## Various Attempts to Define Digital Biomarkers

Our following short overview on various attempts to define digital biomarkers show that according to several scientists such markers describe (patterns of) digital footprints providing insights into biological variables of the human body. This definition is not all-encompassing, because, and for instance, Piau et al. (19) define in their review investigating digital biomarkers in mild cognitive impairment/Alzheimer's disease that digital biomarkers are “objective, quantifiable, physiological, and behavioral data that are collected and measured by means of digital devices, such as embedded environmental sensors, portables, wearables, implantables, or digestibles” (p. 2). Such a definition might present the broadest way to define a digital biomarker, because one could argue that behavioral data in itself is not a biological variable (and the term digital biomarker explicitly hints toward biological variables). On the other hand, one could argue that behavior is an outcome of a biological system and in so far could be seen at least indirectly as a biological variable. In other words, behavior arises from the human body including the human brain, hence we are speaking of a biological system behaving in a certain way. In this realm, we also believe the work of Dagum (16) to be of interest, as it links objectively recorded digital footprints from smartphone interactions to individual differences in neuropsychological test performance. As similar test performances have been linked to diverse brain data [such as *via* MRI correlates; (20–22)], one could make a case to speak in the context of such behavioral measures of digital biomarkers, when such brain data could be (indirectly) inferred from studying digital footprints.

Others might see the term digital biomarker only to be appropriate when (clinical) research establishes a link from the digital signals to direct biological signals or even biological data. For instance, Bent et al. (23) explain in their work that digital biomarkers are “digitally collected data from BioMeTs (e.g., glucose levels) from a continuous glucose monitor (CGM) that are transformed into indicators of health outcomes (e.g., diabetic state)” (p. 1).^1^ Of note, BioMeTs is the abbreviation for Biometric Monitoring Technologies. In a newer work Bent et al. (24) mention that “Digital biomarkers are digitally collected data (e.g., a heart rate biosignal from a wrist-worn wearable) that are transformed into indicators of health outcomes (e.g., risk of cardiovascular disease)” (p. 1). In both defining sentences clearly the link between digital and biological data is obvious, although it is debatable if the biological variable needs to be directly analyzed or if its sufficient when the biological state of the human organism can be predicted in a reliable and valid way via a third (non-biological) variable. To illustrate this: health outcomes (of the human biological system) cannot only be predicted from biological variables, but they can also be inferred via self-report. In the latter situation, the healthcare professional relies at least in part on the subjective information coming from the patient to guide the medical diagnosis. Perhaps it comes not as a surprise then, that in an important paper from 4 years ago, Wright et al. (25) refer to another broad definition stating that digital biomarkers are “consumer-generated physiological and behavioral measures collected through connected digital tools” (p. 155), a medically naïve definition they found online.^2^

Against such a broad understanding of digital biomarkers in many papers, for us the question still arises if some digital biomarkers are more representative than others? In particular when one would search for the “prototype” of a digital biomaker? Nam et al. (26) write in their paper that “The most representative examples of a digital biomarker include heart rate, physical activity and steps measured using a smart band or smart watch” (p. 706).^3^ Beyond that, they write: “In a broad sense, digital biomarker include all human data that can be measured using digital tool” (p. 706). The latter sentence again underlines the view of many scientists to have an expansive view regarding what can be understood when speaking of a digital biomarker. Unfortunately, while prevalent, this view reduces digital biomarkers to digital data acquisition and relieves the scientist from demonstrating value either as a direct measure of a biological process or as a surrogate to a biological endpoint. Seen this way not only human behavior in terms of steps or daily activity might be a digital biomarker, but also when a person fills in a digital questionnaire as this ultimately is also the output of a biological system. Thus, one could indeed say as in Nam et al. (26) that digital biomarkers comprise “all human data than can be measured using digital tool” (p. 706), but from our perspective this definition is unsatisfying in that it fails to connect that data into information on relevant biological processes that would drive clinical decision making. This debate demonstrates that a satisfying definition of the term digital biomarker might also be linked to the function it serves in different research and applied settings, an idea we will revisit at the end of this work.

In sum, the present selection of recent definitions makes it clear, that digital biomarkers are sometimes understood in a narrower or in a broader sense. For researchers new to the field, but also for those being in the field for several years now, this might cause confusion or elicit expectations regarding the methodology used in a paper, when they are confronted with the term digital biomarkers in the title of a paper. Without a clear concept of the term digital biomarker, we fear that research in this area loses focus, whereas systematic taxonomies and clear definitions can help to structure research and bring research findings together in a meaningful way. A clarification of terminology will likely accelerate progress of scientific research using digital biomarkers by avoiding confusion created by blurry and idiosyncratic concepts of digital biomarkers.

Given the large number of papers published each year, in particular in a vivid research area as the present one, we are of the opinion that from an economic perspective it is also of importance to best characterize what one is doing in research. It is not only the authors of a paper, but also the reviewers of manuscripts, who might stumble upon papers with misleading titles or keywords, only at first sight falling in the realm of a given research field. Again, this costs time, an important resource in the scientific community.

We are convinced that these are not the only reasons to be more precise with the terminology used in this area. Many fitting papers for the field of digital biomarkers (regardless of being direct or indirect digital biomarkers) do not even mention the term “digital biomarker” in their work. For instance, when Montag et al. (28) published work linking gray matter volume of the nucleus accumbens to Facebook-log data on the smartphone, they were not aware of the existence of this term. In fact, there is research available that is of relevance for a given field, but such a piece is simply hard to find by some audiences. Of note, other more recent work investigating links between resting-state brain data and smartphone log-data from our perspective would also fit with the “digital-biomarker-discussion” in the present paper, but again the term “digital biomarker” cannot be found in the respective articles [but the term biomarker may be found; see (29, 30)]. Again, we are of the opinion that ambiguous or non-comprehensive terminology hinders progress in the field and might even lead to a waste of research money when research, which has been already carried out in this costly research area, is not discovered in the literature (because of not searching for the right keywords) and is carried out again and again by independent work groups. Of course the replication crisis (31) embraces replication work, but is best when it aligns perfectly with the initial study design of the original paper published—and a perfect replication is unlikely to occur without not adequately knowing what others have done in their research.

## Discussion: Researchers Should Make Clearer in Abstracts and Titles What They Investigate

The overview on the different views regarding the definition of digital biomarkers as presented in the literature from our perspective shows that there is currently a lack of consensus. Each of the mentioned definitions make valid points and we do not want to judge each of them to be wrong or right. Despite this, we believe it to be important to be more precise when labeling one's own work in this field in the future. This starts with the title of the paper, touches the abstract and also the keywords (and of course the terminology used throughout a scientific work). Given the term *digital BIOmarker*, we propose that we should only speak of a digital biomarker, when from digital footprints a clinically accepted or useful biomarker can be directly and objectively sensed. From a language aspect, we believe this to be most fitting, but this proposal without doubt represents also a narrow definition (see also Figure 1). Before we come to our own final conclusion, we believe it to be helpful to travel a bit further in the past and shortly revisit important definitions regarding the term “biomarker” without taking into account its “digital”-companion.

**Figure 1:**
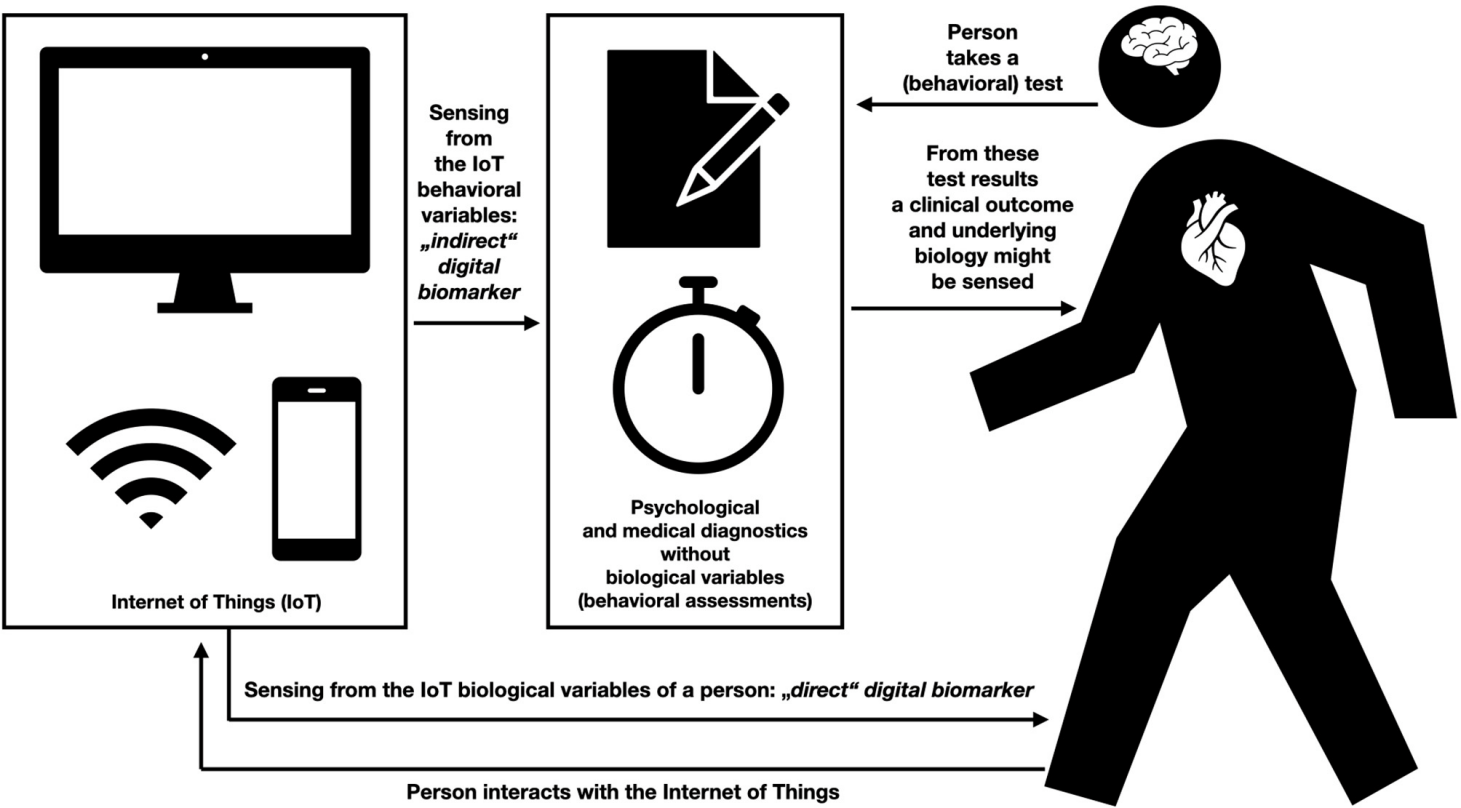
Distinguishing between direct and indirect digital biomarkers.

In a joint sponsorship of the International Labour Organization, the United Nations Environment Programme, and the World Health Organization^4^ it has been put forward that “A biomarker is any substance, structure or process that can be measured in the body or its products and influence or predict the incidence of outcome or disease.”^5^ According to the *Biomarkers Definitions Working Group* the term biomarkers should not only be linked to a clinical outcome and they defined biomarker as “A characteristic that is objectively measured and evaluated as an indicator of normal biological processes, pathogenic processes, or pharmacologic responses to a therapeutic intervention”, p. 91 (32). About 20 years after these statements without doubt we would include certain molecular genetic or epigenetic data, but also endocrinological or immunological data as belonging to the group of biomarkers, in particular when they can predict a biological (health) outcome of relevance (often, but not only clinical). Referring to the word “process” in both definitions we would personally also include the recordings of peripheral-physiological data such as blood pressure, heart rate or electromyography to fall in the realm of biological variables, although clearly additional variables such as eye-movement and other motoric tasks easily could be seen as a behavioral variable and borderline cases. Of interest for the present discussion on what to include or not include when speaking of a digital biomarker, the work by Califf (33) on biomarkers is helpful, where he proposes: “For the sake of clarity, biomarkers should be distinct from direct measures of how a person feels, functions, or survives—a category of measure known as clinical outcome assessment (COA)” (p. 214). On the other hand, this does not suggest to neglect self-report data in the context of biomarker-discussions. For instance, Freedman et al. (34) argue with their work that “including biomarker data in addition to the usual dietary data in a cohort could greatly strengthen the investigation of diet-disease relationships” (p. 1). In their study from nutritional epidemiology, they make a case that the combination of biomarker data *together* with self-report data might in particular be of importance, when the diet-disease relationship is not fully mediated by a biomarker.

With the more classic literature providing definitions of biomarkers, we revisit the current problem of finding consensus regarding the actual nature of a digital biomarker. Here we would like to look at an example, which might be helpful: A meaningful diagnostic biomarker (not digital biomarker) might be a genetic sequence providing insights into who is carrier or non-carrier of Huntington's disease, because this is an autosomal dominantly inherited disease (35). Now imagine, that a study would be able to show that it is possible to predict if a person is carrying the genetic risk for Huntington's Disease *via* digital footprints only. Would these digital footprints then fall in the category of a digital biomarker? In a scenario where both the genetic marker and digital footprints would equally well-predict being carrier vs. non-carrier of Huntington's this might be reasonable (as one then would also make an equivalent prediction from the digital footprints regarding the genetic biomarker). But we believe it is important to be more careful, because in many current works in the realm of digital phenotyping and mobile sensing biological output variables such as mood or other affective states are assessed—and not biological variables (36–38). In the words of Robert Califf (see above) these might be better characterized as COAs. Therefore, and in particular for those working in the field of mobile sensing and digital phenotyping, we suggest that results from neuropsychological tests and from filling in self-report inventories at the moment represent variables fulfilling least the criteria of digital biomarkers in the prototypical sense. If we apply this approach to dementia research in the field of “direct digital biomarkers,” this might mean that digital footprints should tap into the biological processes underlying dementia [such as “disruption of the cholinergic system of the brain,” (39), p. 5], but perhaps not the “visible” psychological functions as measured via memory test performance and so forth. If one still wants to speak of a digital biomarker in the latter case, it might be wise to at least characterize it as an indirect digital biomarker (as behavior is a product of a biological system) or a digital biomarker in a (very) broad sense (see Figure 1). Results that rely on objective behavioral measures, for example passively acquired reaction times to measure the integrity of neural circuits for processing speed or set-shifting/executive function (16), to identify a neurodegenerative disorder can fulfill the notion of a direct digital biomarker, but before this terminology is applied, the links to the relevant biological variables need to be established, something we discuss in more detail below (see that Califf also speaks of individual differences in functioning to belong to COAs).

Of course, the study of digital footprints in psychological and medical sciences all aim at improving the human condition, hence a biological condition. Nevertheless, for reasons of clarity in the scientific exchange the term digital biomarker at the moment might be best used when making direct links between human biology and digital footprints. Beyond this there is always the option when investigating links between digital footprints and psychological/psychiatric variables to speak in general of digital phenotyping/mobile sensing as broader umbrella terms to appropriately describe one's own approach in a given study.

Hence, before we speak of a digital biomarker, a study should bring the distinct proof that from a (pattern of) digital footprint(s) a biological variable or biological process such as direct recorded heart rate actually can be reliably predicted. This means that in studies aiming to find proof for the sound prediction of a biological state from digital footprints on the one side digital footprints need to be recorded via smartphones, wearables or other sources of the Internet of Things and on the other side biological variables need to be investigated. Recent years have shown that this is possible. For instance, digital footprints recorded from smartphones could already be linked to molecular genetics, MRI or PET data footprints (28, 40, 41), but it is also true that the findings from these studies present correlations and to infer causality in such variable sets will be a tremendous task for several years. Hence, before digital biomarkers (hence patterns of digital footprints) alone can be seen as real surrogates for a biological outcome, sophisticated research plans need to be followed and the Clinical Trials Transformation Initiative in June 2017 has made some recommendations to reach such an ambitious goal.^6^

At the end we also wish to name limitations of our work. The main focus of this paper was to highlight the blurry boundaries between various definitions of digital biomarkers in the field and a call for greater precision, in terms of predicting an actual biological variable from the digital footprints. As has been reported in the highly important work by Califf (33) introducing various types of biomarkers, ultimately we might also need to come up with different classes of digital biomarkers ranging from diagnostic, monitoring, pharmacodynamic, predictive, prognostic to safety and susceptibility digital biomarkers, depending on their unique individual functions. In short, digital biomarkers ultimately can and will have different functions when insights are given from digital footprints into human biology. Such different functions will likely have an impact on defining the term digital biomarker appropriately. If a digital biomarker is used for diagnostics (e.g., the example from genetic counseling on Huntington's), it is clear that it needs to be proven that the digital biomarker itself predicts being a carrier vs. non-carrier of the disease in the same way as the genetic analysis itself. If the pattern of digital footprints is proven to be as valid and reliable as the genetic analysis, then we can speak of a direct digital biomarker. Such scenarios without doubt will also depend on effect sizes. In the mentioned example, we observe a monogenetically inherited phenotype, hence in our example carrying a certain genotype results in the outbreak of a (brain) disorder. In other areas, we might have seen for instance that a neuropsychological test correlated with brain activity in a certain region around *r* = 0.30. If a pattern of digital footprints also would be able to establish equal or higher relations with the brain activity (in the context of similar psychological functions), this then might represent the statistical barrier to speak of a digital biomarker. When such a relationship has been established (and has been replicated), (the pattern) of digital footprints alone can be called a digital biomarker.

The present work has not taken this complexity into account as the field is still in its infancy and deals with more obvious problems, perhaps easily summed up with the question: How much biology needs to be in a digital biomarker? One can easily derive from these so far neglected aspects that the study of “real” digital biomarkers from our perspective has just begun.

## Author Contributions

CM wrote the first draft of this manuscript, which was critically revised by JDE and PD. All authors contributed to the article and approved the submitted version.

## Conflict of Interest

CM mentions that he has received (to Ulm University and earlier University of Bonn) grants from agencies such as the German Research Foundation (DFG). CM has performed grant reviews for several agencies; has edited journal sections and articles; has given academic lectures in clinical or scientific venues or companies; and has generated books or book chapters for publishers of mental health texts. For some of these activities he received royalties, but never from gaming or social media companies. CM mentions that he is part of a discussion circle (Digitalität und Verantwortung: https://about.fb.com/de/news/h/gespraechskreis-digitalitaet-und-verantwortung/) debating ethical questions linked to social media, digitalization and society/democracy at Facebook. In this context, he receives no salary for his activities. Finally, he mentions that he currently functions as independent scientist on the scientific advisory board of the Nymphenburg group. This activity is financially compensated. At the moment he also receives funding from a research project on digital phenotyping by Mindstrong Health, Mountain View, CA, USA. JDE notes that he receives royalties for several books published on posttraumatic stress disorder (PTSD); is a paid, full-time faculty member at University of Toledo; occasionally serves as a paid, expert witness on PTSD legal cases; and receives grant research funding from the U.S. National Institutes of Health. PD is the founder of Mindstrong Health, a company developing digital phenotyping for mental healthcare delivery. He served as its Chief Executive Officer from its founding in 2013 through October 2019 and was granted five U.S. patents on digital phenotyping and digital biomarkers. PD is currently co-founder and CEO of Applied Cognition developing diagnostic and therapeutic solutions for Alzheimer's disease. PD owns stock in Mindstrong Health and in Applied Cognition.

## Publisher's Note

All claims expressed in this article are solely those of the authors and do not necessarily represent those of their affiliated organizations, or those of the publisher, the editors and the reviewers. Any product that may be evaluated in this article, or claim that may be made by its manufacturer, is not guaranteed or endorsed by the publisher.

## Footnotes

^1^The mentioned diabetes field is just exemplary of course.

^2^https://rockhealth.com/reports/the-emerging-influence-of-digital-biomarkers-on-healthcare/ (accessed on 6th July 2021).

^3^Please note, that they refer in this sentence to the work: Haghi et al. (27).

^4^In the context of the “framework of the Inter-Organization Programme for the Sound Management of Chemicals” (see also footnote 5).

^5^http://www.inchem.org/documents/ehc/ehc/ehc222.htm (accessed on 15th September 2021).

^6^https://ctti-clinicaltrials.org/wp-content/uploads/2021/06/CTTI_Novel_Endpoints_Recs.pdf (accessed on 15th September 2021).
